# Value of Transnasal Esophagoscopy in the Workup of Laryngo-Pharyngeal Reflux

**DOI:** 10.3390/jcm10143188

**Published:** 2021-07-20

**Authors:** Lukas Horvath, Karolos Fostiropoulos, Emanuel Burri, Marcel Kraft

**Affiliations:** 1 Department of Otorhinolaryngology, Head and Neck Surgery, University Hospital of Basel, 4031 Basel, Switzerland; 2 HNO-Zentrum Beider Basel, 4142 Münchenstein, Switzerland; karolos.fostiropoulos@hirslanden.ch (K.F.); marcel.kraft@unibas.ch (M.K.); 3 Department of Gastroenterology and Hepatology, University Medical Clinic, Kantonsspital Baselland, 4410 Liestal, Switzerland; emanuel.burri@ksbl.ch

**Keywords:** endoscopy, esophagoscopy, extraesophageal reflux, gastroesophageal reflux, nose, deglutition disorders

## Abstract

Background: Laryngopharyngeal reflux (LPR) can display a variety of symptoms, and upper endoscopy is occasionally used for its investigation. The aim of the present study was to determine the value of transnasal esophagoscopy (TNE) in the workup of LPR. Methods: In 200 consecutive patients with suspected LPR, reflux symptom index (RSI), reflux finding score (RFS), oropharyngeal pH-monitoring (PHM) and transnasal esophagoscopy (TNE) were carried out and rated according to the Horvath Score. Results: In the investigation of LPR, TNE showed a sensitivity, specificity and accuracy of 96%, 85% and 95%, respectively. The most common pathologic TNE findings in LPR patients were an insufficient cardia, hiatal hernia, lymphoid follicles and visible reflux. Conclusions: TNE is a supportive method in the workup of LPR, which can display the underlying pathology and directly affect therapeutic decisions.

## 1. Introduction

Laryngopharyngeal reflux (LPR) differs from classic gastroesophageal reflux (GER) in various aspects and has been recognized as an independent clinical entity [1]. These aspects include pathologic mechanisms, symptoms of disease, clinical appearance and therapeutic response [2]. In general, GER presents with lower esophageal sphincter insufficiency, whereas LPR additionally shows a dysfunction of the upper esophageal sphincter [1]. Moreover, GER displays a prolonged acid exposure limited to the esophagus, protracted esophageal clearance and dysmotility, while LPR has none of the aforementioned features. While GER typically presents with heartburn and acid regurgitation, LPR shows a different clinical picture. Nevertheless, it remains difficult to distinguish these two entities, which can also coexist [3,4]. Today, esophagogastro-duodenoscopy (EGD) in the sedated patient and traditional impedance pH-metry are generally used to assess GER [5]. On the other hand, the diagnosis of LPR is frequently based on the reflux symptom index (RSI), reflux finding score (RFS) and/or a trial of proton pump inhibitor (PPI) therapy [6,7]. Recently, we described a completely new approach suggesting four different methods to establish the diagnosis of LPR, because no single test alone is able to reliably confirm or exclude LPR nor to visualize the underlying pathology leading to LPR [8]. Thus, upper endoscopy seems to be a reasonable supplement in the investigation of LPR. Transnasal esophagoscopy (TNE) has been first reported in 1994 describing its feasibility for upper endoscopy [9]. In recent years, TNE has gained popularity among clinicians and patients for both screening and procedural purposes [10]. Studies uniformly agree that this procedure has a diagnostic accuracy to detect GER, which is equal to that of EGD [11,12]. Besides esophageal and extraesophageal reflux disease, TNE makes an interesting tool for assessing mucosal inflammation, scar formation, vascular anomalies, hypopharyngeal diverticula and esophageal neoplasms as well as the follow up of head and neck cancer. In such a manner, the clinician is able to visualize all necessary anatomic areas from the nasal cavity to the stomach under physiological conditions, and the procedure is time- and cost efficient bearing minimal periinterventional risks [10]. TNE is performed as an outpatient procedure and does not need sedation nor general anesthesia. The aim of the present study was to determine the value of this method in the workup of LPR.

## 2. Materials and Methods

A total of 200 patients with suspected LPR, which were consecutively seen at our clinic, were included in our study, using the same inclusion criteria as previously reported [8]. The latter were nonspecific symptoms suspicous for LPR and clinical finidings such as globus sensation, throat clearing, increased mucus, varying hoarseness, dry cough, difficulty swallowing, heartburn as well as posterior laryngitis, laryngeal erythema, thick endolaryngeal mucus, laryngeal oedema and granulation tissue.

The Horvath Score served as the final diagnostic criterion for LPR, and thus oropharyngeal pH-monitoring, RSI, RFS and TNE were carried out for further investigation and then classified by the Horvath Score as depicted in detail elsewhere [8]. In short, this score uses the above mentioned four validated diagnostic methods to categorize LPR into five severity levels or exclude LPR. A detailed description about the performance of oropharyngeal pH-monitoring has previously been discussed [13]. If the Horvath Score was positive, patients of our study underwent antireflux therapy. Accordingly, our study protocol fulfilled the ethical standards of the Declaration of Helsinki.

### 2.1. Transnasal Esophagoscopy (TNE)

**Figure 1:**
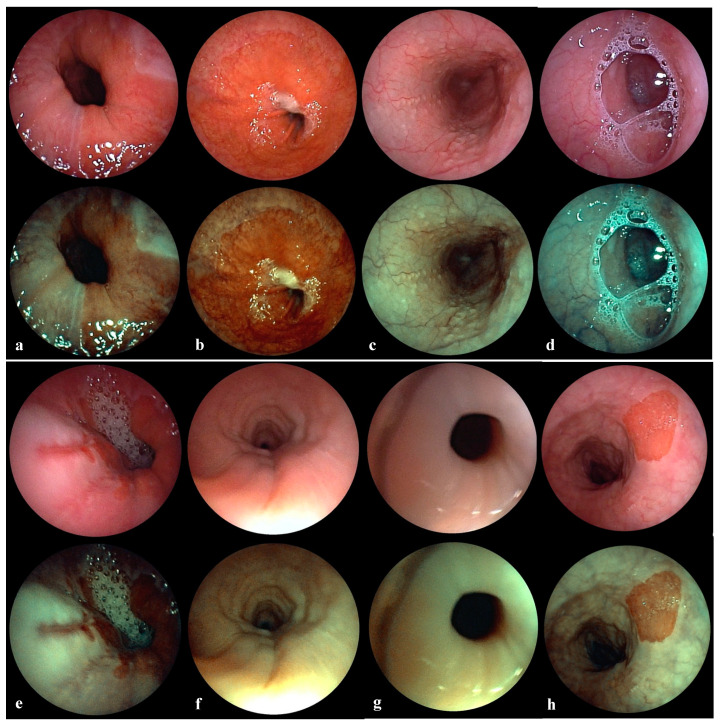
(**a**) Insufficient cardia. White light endoscopy (upper image) demonstrates an insufficient lower esophageal sphincter with a gaping cardia. Chromoendoscopy (lower image) shows gastric mucosa in brown and esophageal mucosa in green thus highlighting the transition zone (Z-line). (**b**) Hiatal hernia. White light endoscopy (upper image) demonstrates a large sliding hiatal hernia after applying abdominal pressure. Chromoendoscopy (lower image) shows gastric mucosa in brown and esophageal mucosa in green thus highlighting the transition zone (Z-line). (**c**) Lymphoid follicles. White light endoscopy (upper image) demonstrates multiple lymphoid follicles along the entire esophagus. Chromoendoscopy (lower image) shows lymphoid tissue in white and dilated vessels in dark green thus highlighting mild mucosal inflammation. (**d**) Visible reflux. White light endoscopy (upper image) demonstrates foamy reflux of stomach contents into the upper esophagus. Chromoendoscopy (lower image) shows lymphoid tissue in white and dilated vessels in dark green thus highlighting mild mucosal inflammation. (**e**) Peptic esophagitis. White light endoscopy (upper image) demonstrates several erosive lesions of the lower esophagus affecting more than one longitudinal fold without confluence (Los Angeles Grade B). Chromoendoscopy (lower image) shows mucosal erosions in dark brown and normal esophageal mucosa in green thus highlighting the inflamed area. (**f**) Tertiary contractions. White light endoscopy (upper image) demonstrates reflux induced uncoordinated contractions of the esophagus. Chromoendoscopy (lower image) clearly shows the circular contraction rings moving esophageal contents in an uncoordinated manner. (**g**) Insufficient upper esophagus sphincter. White light endoscopy (upper image) demonstrates an insufficient upper esophageal sphincter with permanent gaping. Chromoendoscopy (lower image) shows the edematous hypopharyngeal and upper esophageal mucosa in green. (**h**) Ectopic gastric mucosa. White light endoscopy (upper image) demonstrates a small island of ectopic gastric mucosa below the upper esophageal sphincter. Chromoendoscopy (lower image) shows gastric mucosa in brown and esophageal mucosa in green, thus highlighting the ectopic gastric mucosa.

Endoscopy was carried out in the awake and fasting patient in an upright position without sedation. This allows the esophagus to unfold as well as the upper (UES) and lower esophageal sphincter (LES) to display authentic function independent of general anesthesia. A 3.8 mm flexible videoendoscope with a 1.2 mm working channel for air insufflation and/or biopsy was used, which is suitable for complete upper panendoscopy (Fujifilm EB-530 P videobronchoscope, Fujifilm Eluxeo 7000 system, Treier Endoscopie, Beromünster, Switzerland). First, Tenaphin spray 1% (tetracaine 10 mg/mL and naphazoline 0.2 mg/mL) was applied into the nose and lidocaine spray 10% into the throat, before the patient was asked to swallow all remaining anesthetic. Then, the lubricated endoscope was introduced transnasally through the wider nasal cavity. Besides assessing the nose, pharynx and larynx, the esophagus, gastroesophageal junction and the stomach were examined. Minimal air was insufflated with a double-balloon for efficient unfolding of the esophagus in case of collapse. A standard protocol was applied to systematically gather pathological findings such as sphincter insufficiency, gaping cardia, hiatal hernia, visible reflux of gastric contents, peptic esophagitis, Barrett esophagus and ectopic gastric mucosa (Figure 1a–h). Additionally, other eye-catching findings were noted such as tertiary contractions and lymphoid follicles along the entire esophagus. A sphincter insufficiency was considered, when the flexible endoscope entered the esophagus without resistance and/or when an intermittent opening of the cardia was noticeable. A gaping cardia was assessed anterograde from the esophagus and retrograde from the stomach by inverting the videoendoscope towards the LES. A hiatal hernia was diagnosed when the stomach protruded 2 cm or more through the esophageal hiatus into the thorax. Visible reflux was evident by observing gaseous or foamy gastric contents in the esophagus. Peptic esophagitis was defined as erosive or nonerosive lesions of the squamous lining and rated according to the Los Angeles Classification [14]. Barrett esophagus was obvious when salmon-pink colored extensions of mucosa above the gastroesophageal junction were present. Ectopic gastric mucosa was recognized as oval salmon-pink colored patch along the esophagus, especially under the UES. Transnasal esophagoscopy was rated pathological, if one or more of the abovementioned findings were present.

### 2.2. Statistics

The calculation of sensitivity, specificity, accuracy, and positive and negative predictive values in detecting LPR was carried out separately for each diagnostic method. Oropharyngeal pH metry and RSI >13 in severe and nonsevere LPR were considered as positive, but negative in no reflux. Reflux finding score >7 was estimated positive in severe and moderate LPR and negative in all other conditions. Transnasal esophagoscopy was rated positive, if characteristic reflux findings were seen (e.g., sphincter insufficiency, hiatal hernia, gaping cardia, visible reflux, peptic esophagitis, Barrett esophagus, ectopic gastric mucosa), whereas ordinary findings were regarded as negative. Fisher’s exact test was applied for statistical analysis. A *p*-value of <0.05 was regarded statistically significant, while *p*-values of <0.01 were considered as highly significant.

## 3. Results

### 3.1. Clinical Data

A total of 103 male (52%) and 97 female participants (48%) were included in our study. The average age at diagnosis was 62 years. One hundred and five (53%) patients showed severe LPR reaching a Horvath Score of 4–5, 35 (18%) subjects displayed moderate and 26 (13%) mild characteristics with a Horvath Score between 2–3, 11 (6%) individuals had neutral pH-values, and 10 (5%) cases were alkaline also achieving a Horvath Score within 2–3, while in 13 (7%) subjects LPR was absent featuring a Horvath Score of 0–1 (Table 1).

### 3.2. Transnasal Esophagoscopy

Characteristic reflux findings were seen in 181 (91%) of our study participants. One hundred and three (98%) of them belonged to the severe LPR group and 76 (93%) to the nonsevere reflux group, whereas only two (15%) cases without LPR showed positive endoscopic findings (Table 1).

The most common pathologic TNE finding was an insufficient cardia in 140 (70%) cases, followed by hiatal hernia in 125 (63%) patients, lymphoid follicles in 95 (48%), visible reflux of gastric contents in 92 (46%) and peptic esophagitis in 55 (28%) individuals. Less common were tertiary contractions in 63 (32%) and an insufficient UES in 39 (20%) of cases. In general, these pathologic TNE findings showed a similar distribution among reflux severities and the different LPR types (Table 2).

Among acidic reflux, patients with severe LPR showed the highest proportion of pathologic endoscopic findings with 103 (98%) cases, followed by moderate and mild LPR with 31 (89%) and 24 (92%) individuals, respectively. Alkaline and neutral reflux had a rate of 100% pathologic TNE findings each in our cohort, while only two patients (15%) without LPR had pathologic endoscopic findings (Table 2). There were two false positive results (peptic esophagitis, lymphoid follicles with tertiary contractions) in cases without LPR and eight false negative results in individuals with LPR (two severe, four moderate and two mild LPR) showing ordinary TNE findings (Table 3).

In the investigation of LPR, TNE revealed a significantly higher sensitivity (96% vs. 74% and 51%) and accuracy (95% vs. 73% and 64%) than RSI and RFS, respectively, and an equal sensitivity (96% vs. 93%) compared to oropharyngeal pH-monitoring. In contrast, the specificity of TNE (85% vs. 54%, 93% and 94%) remained similar in comparison to RSI, RFS and oropharyngeal pH-monitoring (Table 4).

## 4. Discussion

This is the first study in the literature to evaluate TNE specifically in the assessment of LPR. Other authors have investigated this method in a variety of disorders, among which LPR played only an ancillary role [15,16,17,18]. This makes a comparison of our results with the literature difficult.

An insufficient cardia and hiatal hernia were the most common pathologic TNE findings, representing a weak LES and allowing refluxate to escape from the stomach. These lesions often occur in combination and represent the main cause of LPR. Consequently, a permanent gaping cardia was mainly found in severe LPR, whereas an intermittent gaping cardia and sliding hernia are often not visible during EGD due to body position and sedation. Visible reflux of gastric contents was also a common finding in our study. Refluxate in patients with LPR is often gaseous (reminiscent of sparkling wine) or foamy and rarely fluid. However, gaseous reflux is frequently overlooked or simply not visible during EGD. Peptic esophagitis occurred in less than a third of our study participants. As liquid acid or pepsin ascending from the stomach with a prolonged exposure is necessary for its development, the highest proportion was found in severe and neutral LPR. Peptic esophagitis is the main consequence of GER, hence it can also be present in patients without LPR (GER only) or in coexisting LPR (GER and LPR). In our opinion, peptic esophagitis does not rule out LPR but is indicative for coexisting GER. The esophagus features much better protective mechanisms against refluxate than the mucosa of the throat. In such a manner, typical gaseous reflux of LPR mainly causes symptoms in the throat, whereas fluid reflux of GER predominantly causes mucosal damage in the lower esophagus.

An insufficient UES is difficult to assess during EGD in sedation due to muscle relaxation. In contrast, the tension of the UES is not impaired during TNE in the awake patient. Therefore, a lack of physiological resistance against introducing the videoendoscope through the UES demonstrates sphincter insufficiency allowing the refluxate to ascend to the throat without hindrance, which was most common in severe and moderate LPR. Lymphoid follicles and tertiary contractions, which are probably an expression of mild esophageal inflammation, were frequently detected in our cohort and seem to be rather a consequence of LPR or can potentially arise also in other pathologies. However, an insufficient cardia or hiatal hernia are clearly pathologic findings and not found in normal subjects. Nevertheless, a weakness of this study is the lack of a control group. Lymphoid follicles can be easily overlooked in EGD and deemed unremarkable, whereas tertiary contractions are generally not visible in sedation or general anesthesia. Both lesions might be the cause of dysphagia in patients with LPR (and also GER) by moving esophageal contents in an uncoordinated manner during swallowing and avoiding adequate esophageal clearance. In contrast, Barrett esophagus and ectopic mucosa were rare pathologic TNE findings, which seem to be merely a consequence but certainly not the cause of LPR. In our series, only a few patients with severe LPR displayed the aforementioned lesions (Table 2). Patients with alkaline and neutral reflux showed the highest proportion of pathologic TNE findings, which can be explained by the highly irritative nature of bile salt and pepsin. Hence, the majority of these cases presented with peptic esophagitis confirming our hypothesis. Severe reflux showed equally a high rate in pathologic TNE findings, followed by mild and moderate reflux disease.

In our study, there were two false positive results in patients without LPR. One of these cases showed a peptic esophagitis and another individual lymphoid follicles and tertiary contractions related to GER. Additionally, there were eight false negative results in patients with LPR showing ordinary TNE findings. Two patients with severe LPR demonstrated only signs of gastritis, while TNE was unremarkable in four cases with moderate LPR and in two individuals with mild LPR. These negative TNE findings could possibly be explained by pure gaseous reflux irritating only the pharynx and not the esophagus. 

In our series, no complications occurred. According to the literature, major complications of TNE are exceedingly rare and self-limiting. Minor complications consisting of vasovagal events or epistaxis occur in less than 3% of interventions, wherefore TNE is deemed to be safer than transoral EGD [10,19]. Future prospective studies are needed to compare TNE in the awake patient with transoral EGD in sedation or general anesthesia in the assessment of LPR.

With the help of TNE, the clinician may examine the awake and cooperating patient in an upright position, inspecting the nasal cavity all the way to the level of the gastroesophageal junction and the stomach. By entering the esophagus, passing the scope through the UES gives the investigator an idea regarding the UES sufficiency by feeling resistance or looseness. During the passage towards the stomach, the esophageal mucosal lining can be evaluated. Furthermore, esophageal contractions, refluxing liquid or gas as well as the tone of the LES can be precisely observed in the awake patient. The LES behaves differently in the absence of sedation or general anesthesia, thus a gaping cardia or sliding hiatal hernia can be uncovered. Additionally, a swallowing examination in cooperation with the patient is possible, and the inner lining of the stomach can be visualized. The scope’s small diameter and local anesthesia allow for better functional evaluation of LPR, swallowing disorders and probably also GER. Furthermore, the ease and safety of TNE has made it valuable, particularly in the care of patients with medical comorbidities or head and neck cancer. TNE appears also suitable to perform saliva sampling directly at the location of reflux damage (e.g., esophagus, pharynx, larynx) to test for pepsin with the Peptest or for contents of bile salts and acidity of secretions. Subsequently, further investigations could be applied to confirm LPR. With precise anatomical knowledge of the nose, pharynx and larynx, TNE can be equally performed by gastroenterologists or otolaryngologists, who are familiar with this technique.

Disadvantages of TNE are the small working channel making multiple biopsies in Barrett esophagus and other endoscopic interventions difficult to perform. Moreover, a strong gag reflex, pronounced discomfort and lack of patient cooperation (e.g., disabled person, children) makes TNE potentially impossible. In these cases, EGD in sedation is probably a better option. When awake TNE displays gastritis, peptic esophagitis and/or Barrett esophagus, EGD is also needed for further investigation and follow-up. Other indications for the latter are alkaline LPR to further assess bile reflux. EGD should equally be performed if antireflux surgery is planned. In these cases, impedance pH-monitoring and high-resolution manometry should additionally be completed.

## 5. Conclusions

Gastric contents can reflux up to the pharynx due to weakness along the esophageal passage, best recognizable in the awake patient. Signs of LPR are local mucosal irritation, anatomical abnormalities and visible reflux. Hence, the most common endoscopic findings in LPR patients are a gaping cardia and sliding hiatal hernia, which are purely functional conditions. Frequently, the esophagus shows only lymphoid follicles and/or tertiary contractions and not peptic esophagitis (except in coexisting GER). Therefore, TNE is a supportive method in the workup of LPR, which can display the underlying pathology and directly affect therapeutic decisions. However, this procedure does not stand alone and has to be interpreted along with other validated diagnostic methods, such as RSI, RFS and oropharyngeal pH-monitoring.

## Figures and Tables

**Table 1:** Study outcome for each approach in detecting LPR conforming to the Horvath Score (*n* = 200).

Horvath Score	Severity	Pathologic RSI	Pathologic RFS	Pathologic PHM	Positive Ryan	Pathologic TNE	Total
4–5	Severe LPR	84 (80%)	63 (60%)	105 (100%)	98 (93%)	103 (98%)	105 (53%)
2–3	Nonsevere LPR	55 (67%)	17 (21%)	79 (96%)	7 (9%)	76 (93%)	82 (41%)
	Moderate	21 (60%)	7 (20%)	33 (94%)	6 (17%)	31 (89%)	35 (18%)
	Mild	17 (65%)	3 (12%)	26 (100%)	1 (4%)	24 (92%)	26 (13%)
	Neutral	10 (91%)	4 (36%)	10 (91%)	0 (0%)	11 (100%)	11 (6%)
	Alkaline	7 (70%)	3 (30%)	10 (100%)	0 (0%)	10 (100%)	10 (5%)
0–1	No LPR	6 (46%)	0 (0%)	0 (0%)	0 (0%)	2 (15%)	13 (7%)

Abbreviations: LPR, laryngopharyngeal reflux; RSI, Reflux Symptom Index; RFS, Reflux Finding Score; PHM, oropharyngeal pH-monitoring; Ryan, Ryan Score; TNE, transnasal esophagoscopy.

**Table 2:** Results of transnasal esophagoscopy in the assessment of laryngopharyngeal reflux (*n* = 200).

TNE	Severe LPR	Moderate LPR	Mild LPR	Neutral LPR	Alkaline LPR	No LPR	Total
Pathologic findings	103 (98%)	31 (89%)	24 (92%)	11 (100%)	10 (100%)	2 (15%)	181 (91%)
Insufficient cardia	79 (75%)	24 (69%)	19 (73%)	10 (91%)	8 (80%)	0 (0%)	140 (70%)
Hiatal hernia	72 (69%)	20 (57%)	16 (62%)	9 (82%)	8 (80%)	0 (0%)	125 (63%)
Lymphoid follicles	48 (46%)	19 (54%)	13 (50%)	8 (73%)	6 (60%)	1 (8%)	95 (48%)
Visible reflux	49 (47%)	19 (54%)	12 (46%)	6 (55%)	5 (50%)	1 (8%)	92 (46%)
Peptic esophagitis	31 (30%)	8 (23%)	5 (19%)	7 (64%)	3 (30%)	1 (8%)	55 (28%)
Tertiary contraction	30 (29%)	13 (37%)	8 (31%)	6 (55%)	5 (50%)	1 (8%)	63 (32%)
Insufficient UES	20 (19%)	10 (29%)	3 (12%)	5 (45%)	1 (10%)	0 (0%)	39 (20%)
Barrett esophagus	3 (3%)	0 (0%)	0 (0%)	0 (0%)	0 (0%)	0 (0%)	3 (2%)
Ectopic mucosa	1 (1%)	0 (0%)	0 (0%)	0 (0%)	0 (0%)	0 (0%)	1 (1%)
Normal findings	2 (2%)	4 (11%)	2 (8%)	0 (0%)	0 (0%)	11 (85%)	19 (9%)

Abbreviations: TNE, transnasal esophagoscopy; LPR, laryngopharyngeal reflux; UES, upper esophageal sphincter.

**Table 3:** Evidence of LPR (*n* = 200).

Method	True Positive	False Positive	False Negative	True Negative	Total
RSI	138	6	49	7	200
RFS	73	4	69	54	200
PHM	98	6	7	89	200
TNE	179	2	8	11	200

Abbreviations: LPR, laryngopharyngeal reflux; RSI, Reflux Symptom Index; RFS, Reflux Finding Score; PHM, oropharyngeal pH-monitoring; TNE, transnasal esophagoscopy.

**Table 4:** Detection of laryngopharyngeal reflux (*n* = 200).

Method	Sensitivity	Specificity	Accuracy	Pos. Pred. Value	Neg. Pred. Value
RSI	74% ^†^ (*p* = 0.000)	54% (*p* = 0.101)	73% ^†^ (*p* = 0.000)	96% (*p* = 0.080)	13% ^†^ (*p* = 0.000)
RFS	51% ^†^ (*p* = 0.000)	93% (*p* = 0.231)	64% ^†^ (*p* = 0.000)	95% (*p* = 0.067)	44% (*p* = 0.186)
PHM	93% (*p* = 0.290)	94% (*p* = 0.193)	94% (*p* = 0.334)	94% * (*p* = 0.029)	93% ^†^ (*p* = 0.000)
TNE	96%	85%	95%	99%	58%

Abbreviations: RSI, Reflux Symptom Index; RFS, Reflux Finding Score; PHM, oropharyngeal pH-monitoring; TNE, transnasal esophagoscopy. Note: Pathologic findings are classified as positive, normal findings as negative for the calculation of sensitivity, specificity, accuracy, and positive and negative predictive values. * Statistical significance (*p* < 0.05); ^†^ high statistical significance (*p* < 0.01).

## Data Availability

The data presented in this study are available on request from the corresponding author. The data are not publicly available due to privacy or ethical restrictions.
